# Quantitative evaluation of statistical errors in small-angle X-ray scattering measurements

**DOI:** 10.1107/S1600576717003077

**Published:** 2017-03-29

**Authors:** Steffen M. Sedlak, Linda K. Bruetzel, Jan Lipfert

**Affiliations:** a Department of Physics, Nanosystems Initiative Munich, and Center for NanoScience, LMU Munich, Amalienstrasse 54, Munich, 80799, Germany

**Keywords:** small-angle X-ray scattering, SAXS, measurement errors, simulations, scattering profiles, hybrid pixel detectors

## Abstract

A new model is proposed for the measurement errors incurred in typical small-angle X-ray scattering (SAXS) experiments, which takes into account the setup geometry and physics of the measurement process. The model accurately captures the experimentally determined errors from a large range of synchrotron and in-house anode-based measurements. Its most general formulation gives for the variance of the buffer-subtracted SAXS intensity σ^2^(*q*) = [*I*(*q*) + const.]/(*kq*), where *I*(*q*) is the scattering intensity as a function of the momentum transfer *q*; *k* and const. are fitting parameters that are characteristic of the experimental setup. The model gives a concrete procedure for calculating realistic measurement errors for simulated SAXS profiles. In addition, the results provide guidelines for optimizing SAXS measurements, which are in line with established procedures for SAXS experiments, and enable a quantitative evaluation of measurement errors.

## Abstract 2

A model is presented for the errors in small-angle X-ray scattering profiles that takes into account the physics of the measurement process. The model agrees quantitatively with the variations observed in experimental measurements and provides a straightforward prescription to add realistic errors to simulated scattering profiles.

## 1. Introduction   

Small-angle X-ray scattering (SAXS) is a powerful technique to probe the structure, conformations and dynamics of biological macromolecules and their complexes in solution (Vachette *et al.*, 2003 ▸; Svergun & Koch, 2003 ▸; Lipfert & Doniach, 2007 ▸; Putnam *et al.*, 2007 ▸; Hura *et al.*, 2009 ▸; Blanchet & Svergun, 2013 ▸). A particular advantage of the SAXS technique is the ability to study macromolecules under virtually arbitrary solution conditions, from (near) physiological to highly denaturing. The ability to probe even complex and/or partially folded macromolecules and their assemblies in solution has made SAXS a popular tool for structural biology. SAXS data are routinely used in increasingly complex analyses, ranging from the traditional Guinier fits (Guinier, 1939 ▸) and regularized Fourier transformations (Glatter, 1977 ▸; Moore, 1980 ▸; Svergun, 1992 ▸), to *ab initio* shape reconstructions of proteins (Svergun, 1999 ▸; Walther *et al.*, 2000 ▸) and nucleic acids (Lipfert, Das *et al.*, 2007 ▸; Lipfert, Chu *et al.*, 2007 ▸), and hybrid methods that incorporate data from a combination of measurement modalities (Grishaev *et al.*, 2005 ▸; Putnam *et al.*, 2007 ▸; Rambo & Tainer, 2013*a* ▸,*b* ▸; Schindler *et al.*, 2016 ▸; Chen & Hub, 2015 ▸; Tuukkanen *et al.*, 2016 ▸; Bernadó *et al.*, 2007 ▸; Konarev *et al.*, 2016 ▸).

**Figure 1:**
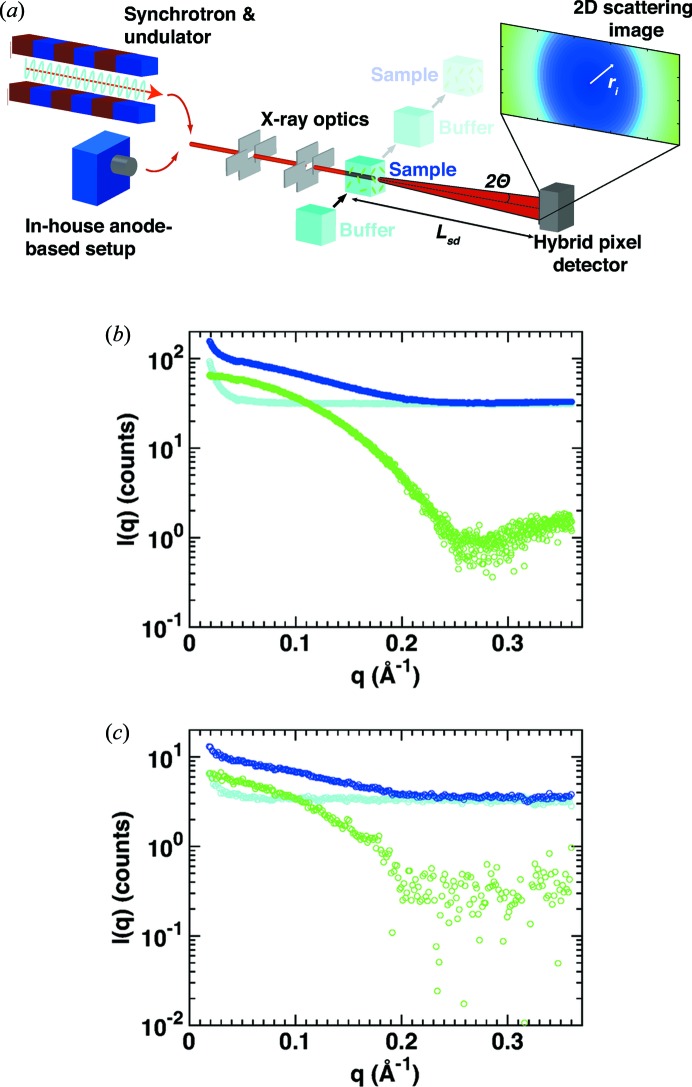
Principle of biological SAXS measurements. (*a*) Schematic of a SAXS setup. At a synchrotron, electrons passing through an undulator (or wiggler or bending magnet) produce X-rays; alternatively an anode source is used at in-house setups. The beam is collimated and directed at a measurement cell filled with either protein sample or buffer only. A hybrid pixel detector records two-dimensional scattering images, which are transformed to one-dimensional scattering profiles. (*b*)–(*c*) One-dimensional scattering profiles from the sample (cytochrome *c* at 8 mg ml^−1^; dark blue) and buffer (light blue) and the resulting buffer-subtracted scattering profiles (green) obtained at (*b*) a synchrotron source (exposure time 1 s; BM29, ESRF, Grenoble) and (*c*) an in-house source (Bruetzel *et al.*, 2016 ▸) (exposure time 2 h; Department of Physics, LMU Munich).

In a typical SAXS measurement, the macromolecules of interest are dissolved in an appropriate buffer and a scattering pattern is recorded (Fig. 1 ▸ *a*). Subsequently, the scattering pattern of pure buffer is measured. After circular averaging of the two scattering patterns, the buffer profile is subtracted from the scattering profile of the macromolecular sample to obtain the final scattering profile, which is used for further processing and analysis (Figs. 1 ▸ *b* and 1 ▸ *c*). In the measurement process a range of systematic and statistical errors are, at least potentially, introduced. Possible sources of measurement error include (i) problems with sample preparation, purification and homogeneity, (ii) radiation damage of the sample during measurement, (iii) scattering contributions from components of the setup such as the sample cell, beamstop and X-ray windows, (iv) the inherent beam divergence, (v) errors due to detector noise and counting statistics, and (vi) errors in buffer subtraction, for example, due to a mismatch in the beam intensity or in buffer composition as well as alterations of the setup between buffer and sample measurements.

Despite the fact that SAXS profiles are applied in increasingly sophisticated analyses, there is currently no widely accepted and tested model for the errors in SAXS profiles. A solid understanding and quantification of the errors in SAXS measurements are desirable for several reasons: (i) to quantify the reliability of SAXS measurements and to assess the goodness of fit of, for example, a model against experimental data; (ii) to quantitatively compare and optimize different setups; (iii) to simulate SAXS profiles including the appropriate noise.

In particular, in the context of simulating SAXS profiles, different models for the error on scattering profiles have been put forward. A popular choice of model for the error on SAXS profiles is to add Gaussian noise to the scattering intensity in every *q* bin with zero mean and a constant standard deviation (Bernadó *et al.*, 2007 ▸; Schindler *et al.*, 2016 ▸; Förster *et al.*, 2008 ▸; Pinfield & Scott, 2014 ▸), which is often expressed as a percentage of the forward scattering intensity *I*(0) or the scattering intensity at the highest scattering angle *I*(*q*_max_). This choice of a constant Gaussian error corresponds to setting the variance σ^2^(*q*) = *a* ^2^, where *a* is a constant. Values for *a* described in the literature (Förster *et al.*, 2008 ▸; Pinfield & Scott, 2014 ▸; Zettl *et al.*, 2016 ▸) range from 0.3*I*(*q*_max_) to 10^−4^ *I*(0). Given that the scattering intensity for biological macromolecules tends to decrease with increasing *q*, the choice of a constant standard deviation for all *q* values corresponds to a (often much) larger relative error at higher *q*. An alternative choice of model is to introduce Gaussian noise with a *q*-dependent standard deviation σ(*q*); one such model (Stovgaard *et al.*, 2010 ▸) proposed the use of σ(*q*) = *I*(*q*)(*q* + α)β with constants α = 0.15 and β = 0.3. A similar model is used by the program *FoXS* to estimate the uncertainties of the scattering intensity when computing a SAXS profile from a crystal structure (Schneidman-Duhovny *et al.*, 2010 ▸), though *FoXS* uses different constants and additionally employs a Poisson distribution. Similarly, the *FoXS* web server (Schneidman-Duhovny *et al.*, 2010 ▸) will assume errors distributed according to a Poisson distribution with an expectation value (which is equal to the variance for Poisson distributions) of 10, unless the user provides an experimental measurement error.

Comparing the models currently described in the literature with experimental data (see Fig. S1 in the supporting information), we find that they fail to quantitatively capture the experimentally observed errors for the entire *q* range. Here, we first derive and then test a new model for the measurement errors in SAXS experiments that provides an accurate description of experimental data over a large range of measurement parameters.

## 2. Materials and methods   

### 2.1. Samples for SAXS measurements   

Cytochrome *c*, lysozyme and bovine serum albuminum (BSA) were purchased from Sigma–Aldrich and applied without further purification. The lyophilized powder of each protein was weighed to prepare a stock solution of the highest concentration and diluted to the required concentrations. Cytochrome *c* was dissolved in 100 m*M* acetate buffer pH 4.6, with 0.5 *M* guanidinium hydrochloride added. For lysozyme a 40 m*M* acetate buffer pH 4.5, with 150 m*M* NaCl added, was prepared. BSA was dissolved in 50 m*M* HEPES pH 7.5, 50 m*M* KCl. Prior to the measurements, both buffer and sample solutions were filtered through 0.22 µm syringe filters (Thermo Scientific, USA) and centrifuged at 13 500 r min^−1^ for 10 min in a tabletop centrifuge (Eppendorf, Germany). For in-house SAXS measurements sample and buffer solutions were degassed in a desiccator at a pressure level of 30 mbar (3 kPa) for 20 min to prevent the formation of air bubbles in the sample chamber during experiments. 80 µl of sample or buffer solution was loaded into the sample chambers. For synchrotron measurements 35 µl of sample or buffer solution was used.

### 2.2. SAXS data acquisition   

In-house SAXS measurements were performed with an Mo GeniX^3D^ microfocus X-ray tube (Xenocs SA, Sassenage, France) combined with FOX2D single reflection optics delivering a monochromatic beam with an X-ray energy of 17.4 keV (Bruetzel *et al.*, 2016 ▸). The sample–detector distance was set to ∼1.11 m, yielding usable *q* values between 0.05 and 0.35 Å^−1^. We used a PILATUS 100K detector (DECTRIS Ltd, Switzerland) for X-ray detection. For each experiment, sample and buffer profiles were collected with three to five exposures of 2 h each.

All synchrotron data, except for the data presented in Figs. 6 and 7, were collected at beamline BM29 at the ESRF in Grenoble at an X-ray energy of 12.5 keV and a sample–detector distance of 2.87 m, resulting in a usable *q* range of 0.05–0.35 Å^−1^ (Pernot *et al.*, 2013 ▸). We used a PILATUS 1M (DECTRIS Ltd, Switzerland) detector for data acquisition. Data were collected in ‘flow’ mode at room temperature with ten measurement frames at an exposure time of between 1 and 4 s in ‘multibunch mode’ or ‘low bunch mode’.

## 3. Results and discussion   

We propose a new model for the errors in a typical SAXS measurement and evaluate the model against measured SAXS data from a range of experimental setups that employ hybrid pixel detectors (Broennimann *et al.*, 2006 ▸; Henrich *et al.*, 2009 ▸). While sample quality and (the absence of) radiation damage are critical factors in any SAXS measurement (Hura *et al.*, 2009 ▸; Jeffries *et al.*, 2016 ▸; Dyer *et al.*, 2014 ▸), they tend to be specific to the sample under investigation (Hopkins & Thorne, 2016 ▸). In this work, we will focus, therefore, on errors that are intrinsic to the SAXS measurement process, *i.e.* statistical errors resulting from photon counting statistics. Systematic errors and radiation damage are not treated further here. We note that the errors considered in our model are unavoidable in any physical measurement and constitute a best-case scenario, which is most relevant for simulations of SAXS profiles. All test measurements reported in this work use well characterized samples that are pure and monodisperse and do not suffer from radiation damage under the measurement conditions employed. Most calibration measurements used cytochrome *c*, a protein typical of weakly scattering biological samples that has been used as a scattering standard previously (Bruetzel *et al.*, 2016 ▸). Data were collected at state-of-the-art synchrotron-based (Pernot *et al.*, 2013 ▸; Lipfert *et al.*, 2006 ▸; Beno *et al.*, 2001 ▸) and in-house anode-based SAXS setups (see §2 for details). We confirmed the absence of radiation damage by partitioning the total exposure time of each measurement into frames and testing for significant changes in the scattering curves in subsequent frames (Fig. S2).

Our new error model is based on the following assumptions: (i) scattering and photon counting are Poisson processes; (ii) the scattering intensity of the buffer profile is approximately constant over the whole *q* range; (iii) buffer and sample measurements have independent statistical errors.

### 3.1. Counting statistics   

Raw SAXS data are two-dimensional images (Fig. S3) providing the number of counts per pixel *n_i_*. To obtain a scattering profile, every pixel is assigned to the appropriate momentum transfer value *q* and the one-dimensional intensities for the sample *I*_s_(*q*) and the buffer *I*_b_(*q*) (in units of counts) are calculated by averaging over all *N*(*q*) pixels belonging to the same *q* bin:
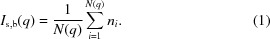
Assuming that the individual pixels have independent statistical errors σ_*i*_, the variance of the intensity (*i.e.* of the sample mean) at a given value of *q* is given by (a detailed derivation is provided in the supporting information)
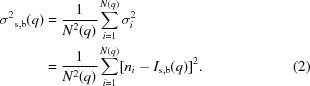
While the second line of equation (2) is applied to evaluate and quantify errors from experimental data, the more general formulation in the first line will be used in the next steps. Assuming that scattering and photon counting are Poisson processes, the mean and the variance of the distribution of counts are equal, which results in
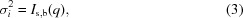


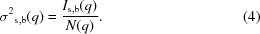



**Figure 2:**
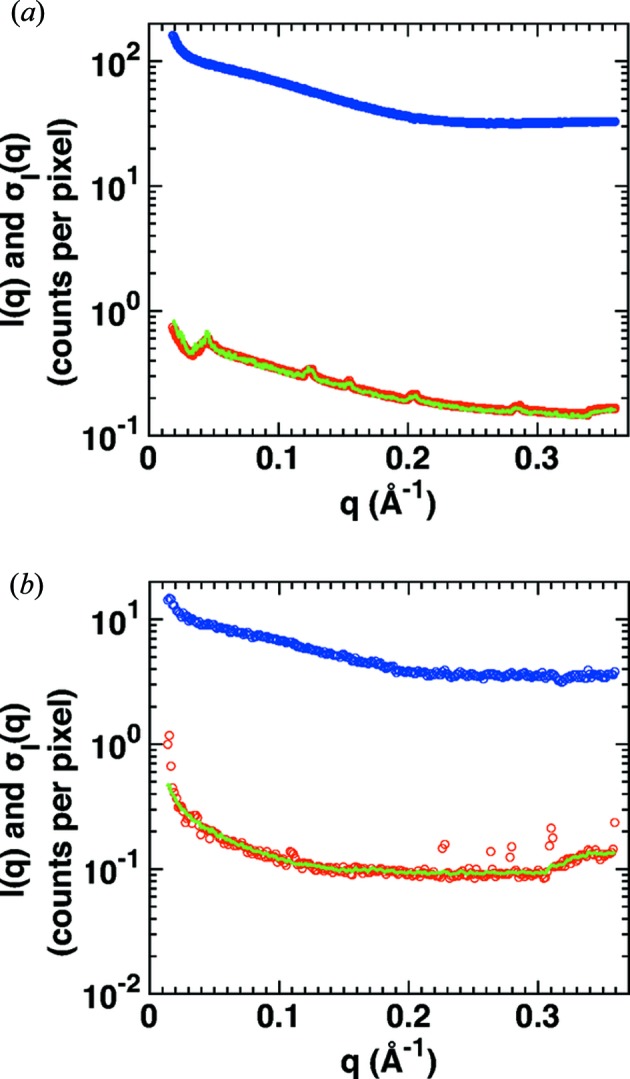
Counting statistics of SAXS profiles before buffer subtraction. SAXS measurements of cytochrome *c* (at 8 mg ml^−1^) at (*a*) a synchrotron source (exposure time 1 s; BM29, ESRF, Grenoble) and (*b*) an in-house source (Bruetzel *et al.*, 2016 ▸) (exposure time 2 h; Department of Physics, LMU Munich). Scattering profiles of the protein samples after circular averaging are shown as blue circles; the corresponding standard errors of the mean σ_s_ computed from the counts in the individual pixels [equation (2)] are shown as red circles. The green line is the square root of the intensity divided by the number of pixels per *q* bin {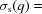 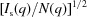}.

We find the relationship in equation (4) to be valid for both in-house and synchrotron data over the entire measured *q* range (Fig. 2 ▸). For the synchrotron data, missing pixels between the different detector modules (Fig. S3) cause small increases in the variance at specific *q* values due to the decreases in the number of pixels in these *q* bins, which are correctly reproduced by the model in equation (4) (Fig. 2 ▸ *a*). For the in-house data, there are some outliers in the variance for large *q*, which result from broken pixels (Fig. 2 ▸ *b*).

### 3.2. Buffer subtraction   

In biological SAXS experiments, a buffer profile is subtracted from the sample profile to obtain the macromolecular scattering curve, which is used for further analysis:
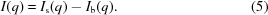
Assuming buffer and sample measurements to be independent, we have to propagate the uncertainties by adding the variances:
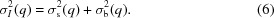



For simplicity, we approximate the buffer profile to be constant over the whole *q* range. This is a good approximation for all but the lowest *q* values (Figs. 1 ▸ *b* and 1 ▸ *c*) and we find that in practice it works well over the entire *q* range considered in our measurements (see below). It is convenient to relate the buffer profile intensity to the sample profile (at an arbitrary *q* value *q*_arb_) by introducing a contrast factor *c*:
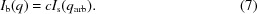
Since the buffer profile is approximately constant for intermediate to large *q* values, *q*_arb_ can be chosen arbitrarily within this constant buffer range (Figs. 1 ▸ *b* and 1 ▸ *c*). The choice of *q*_arb_ then sets the values of *I*_s_(*q*_arb_) and *c*, such that the *cI*_s_(*q*_arb_) is constant, and defines the level of the constant buffer intensity. Equations (4), (5) and (7) allow us to rewrite equation (6) as (a step-by-step derivation can be found in the supporting information)
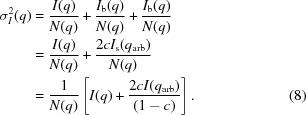



The second term in the sum in equation (8) represents the constant buffer intensity. We note that it is constant and independent of *q*_arb_. The choice of *q*_arb_ affects the values of *c* and *I*(*q*_arb_), but not the overall value of the second term. From an experimental point of view, it might appear unnecessarily complicated to not keep the buffer and sample intensities explicitly; however, for simulations of experimental noise for computed scattering profiles the formulation of equation (8) is very convenient. Typical calculations of theoretical SAXS profiles [*e.g.* using *CRYSOL* (Svergun *et al.*, 1995 ▸) or *FoXS* (Schneidman-Duhovny *et al.*, 2010 ▸)] from crystal structures do not generate separate buffer *I*_b_(*q*) and sample *I*_s_(*q*) scattering profiles and only the final intensity *I*(*q*) is provided. Therefore, the form of equation (8) is advantageous, because it only contains *I*(*q*), *N*(*q*) and *c*. The number of pixels per *q* bin *N*(*q*) can be approximated as shown in the following section. The contrast *c* between sample and buffer intensity at a certain, arbitrary, scattering vector *q*_arb_ has to be estimated and we provide typical values derived from experimental data in Table 1 ▸ and Table S1 in the supporting information.

### 3.3. Effects of the setup geometry   

Equation (8) states an inverse proportionality of the variance and the number of pixels per *q* bin, which in turn is determined by the setup and detector geometry. Especially for low count rates and small detector dimensions, frequently encountered at in-house setups, the setup geometry is of great importance to achieve good data quality. The exact binning of pixels is subject to some freedom, but for a standard SAXS geometry where the detector is placed orthogonally to the beam and the *q* range is linear, *i.e.* the size of the *q* bins is constant, one finds
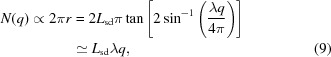
where *L*_sd_ is the sample–detector distance, *r* the distance of a pixel to the beam centre on the detector and λ the X-ray wavelength. This geometrical relation is illustrated in Fig. 1 ▸(*a*). We use the convention of 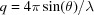 for the absolute value of the momentum transfer *q*, where 2θ is the angle between the incident and the scattered beam.

**Figure 3:**
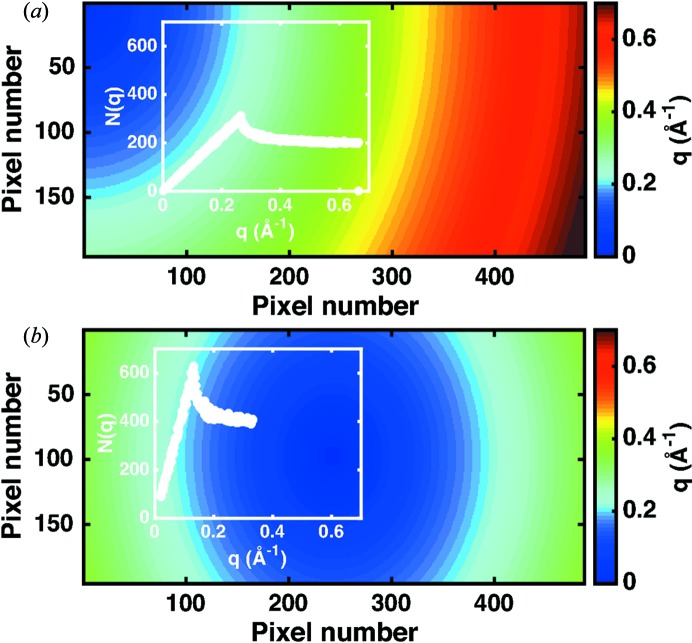
Measurement geometry and the number of pixels per *q* bin. Assignment of the pixels in two dimensions to *q* bins for different positions of the beam centre on a PILATUS 100K detector, with an X-ray energy of 17.4 keV and a sample–detector distance of 1.11 m. (*a*) Configuration with the beam centre aligned on the top left corner of the detector. (*b*) Setup with the beam centred on the detector. The insets show the resulting pixels per *q* bin *N*(*q*). Further beam centre positions are depicted in Fig. S4.

The detector dimensions restrict the values for *r*. For small detectors, *r* is tightly confined and the exact setup geometry is relevant for the number of pixels per *q* bin. By varying the sample–detector distance and/or the position of the beam centre on the detector, the available *q* range and the number of pixels per *q* bin (and thus the variance) can be changed (Fig. 3 ▸ and Figs. S3–S5). For larger detectors, *r* is less constrained for the same *q* range. In general, the number of pixels per *q* bin *N*(*q*) can approximated by 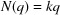, at least for small scattering angles, so that
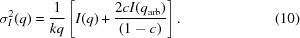



Comparison with experimental data allows for determination of *c* and *k*. The value of *c* will depend on the choice of *q*_arb_ and on the sample. In contrast, *k* depends predominantly on the setup geometry.

### 3.4. Measurement errors for in-house and synchrotron measurements   

**Figure 4:**
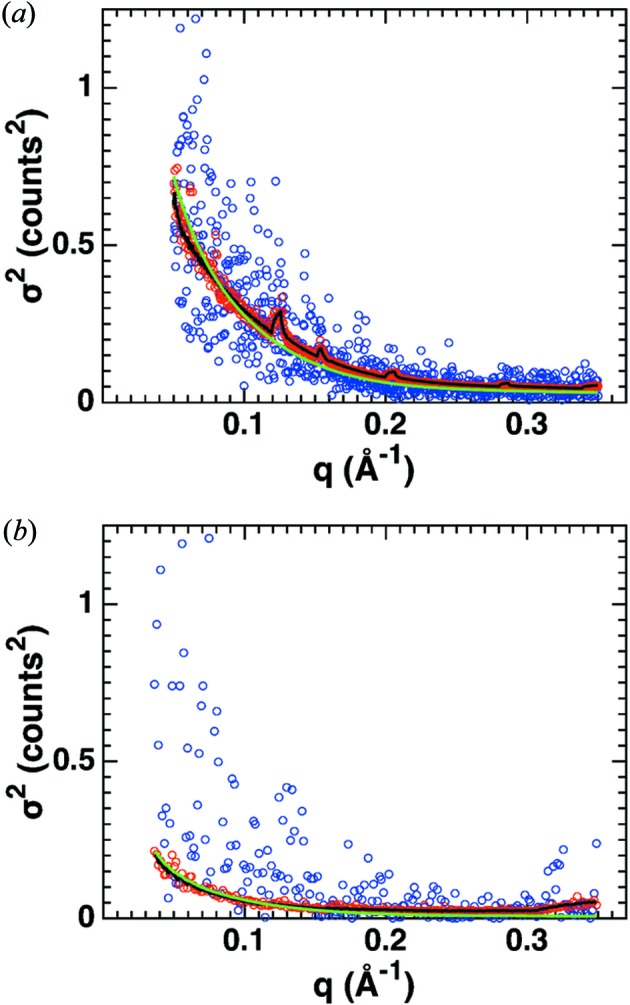
SAXS measurement errors after buffer subtraction. SAXS measurement of cytochrome *c* (24 mg ml^−1^) obtained (*a*) at a sychrotron source (BM29, ESRF, Grenoble) and (*b*) from our in-house source (Department of Physics, LMU Munich). The propagated standard errors for the buffer-subtracted measurement from single exposures of sample and buffer are shown as red symbols. The black lines are co-plots of equation (8) with the red data, using the exact number of pixels per *q* bin for the respective setups and determining the contrast factor *c* by dividing the buffer by the sample intensity at *q*_arb_ = 0.2 Å^−1^ [synchrotron: *c* = 0.69, *I*(*q*_arb_) = 14.20; in-house setup: *c* = 0.68, *I*(*q*_arb_) = 2.30]. The green lines show fits of equation (10) to the data, with *k* as a free fitting parameter (synchrotron: *k* = 6104; in-house setup: *k* = 4298). The blue data points show the variance in the intensity determined from repeat exposures (synchrotron: ten exposures of 4 s each; in-house setup: three exposures of 3 h each) for comparison.

First, we consider a single sample frame and a single buffer frame only (using 24 mg ml^−1^ cytochrome *c* as a representative test sample; red circles in Fig. 4 ▸). The final scattering profile *I*(*q*) and the corresponding variances σ^2^(*q*) are calculated by circular averaging of the pixels for both the sample and buffer profiles and subsequent buffer subtraction while propagating the errors, according to equations (2), (5) and (6). We compare the experimental data with our model using the exact *N*(*q*) [equation (8); black line in Fig. 4 ▸] as well as the approximation [equation (10); green line in Fig. 4 ▸] with *q*_arb_ = 0.2 Å^−1^. As described in §3.2, the value for *q*_arb_ can be chosen arbitrarily. Our choice of *q*_arb_ = 0.2 Å^−1^ is motivated by the observation that for smaller scattering angles the assumption of constant buffer intensity becomes inaccurate while for larger angles the number of pixels per *q* bin decreases and thus the measurement errors in *I*_b_ and *I*_s_ increase. Since *I*(*q*_arb_) and *c* are determined from the sample and buffer scattering intensities at *q*_arb_, there are no free parameters if the exact number of pixels per *q* bin is used; if the approximation in equation (10) is used, the only free fitting parameter is *k*. We find an excellent agreement between our model and the experimental data if the exact number of pixels per *q* bin is taken into account (compare red data and black lines in Fig. 4 ▸). For the synchrotron data a good fit is achieved even if the approximation for the number of pixels per *q* bin is used [equation (10); green line in Fig. 4 ▸(*a*)]. For the in-house data, if the linear approximation for the number of pixels per *q* bin [equation (10)] is used, the fit still captures the right trend and magnitude, but is less convincing. Consequently, it is preferable to use the exact number of pixels per *q* bin [equation (8)] for in-house data, mostly owing to the smaller detector.

As an alternative way to estimate the measurement errors, we computed the variances of the buffer-subtracted scattering intensities from repeat exposures (blue dots in Fig. 4 ▸). We first perform circular averaging on every single sample and buffer frame. Then, we form pairs of sample and buffer profiles. Using equation (5), we calculate a final scattering profile for each pair. Now, we determine the variances between the resulting scattering intensities in each *q* bin. We find the variances computed from repeat exposures to be distributed more broadly compared with the errors estimated from single frames (Fig. 4 ▸, compare blue to red points), which is likely to be due to the still comparatively low number of frames. Note that with a larger number of frames the variance from the repeated exposures more closely resembles the estimate from one pair of frames (compare Figs. 4 ▸ *a* and 4 ▸ *b*). Importantly, the functional dependence of the variance on *q* is very similar for the two estimates.

### 3.5. Experimental errors and optimization of SAXS measurements   

**Figure 5:**
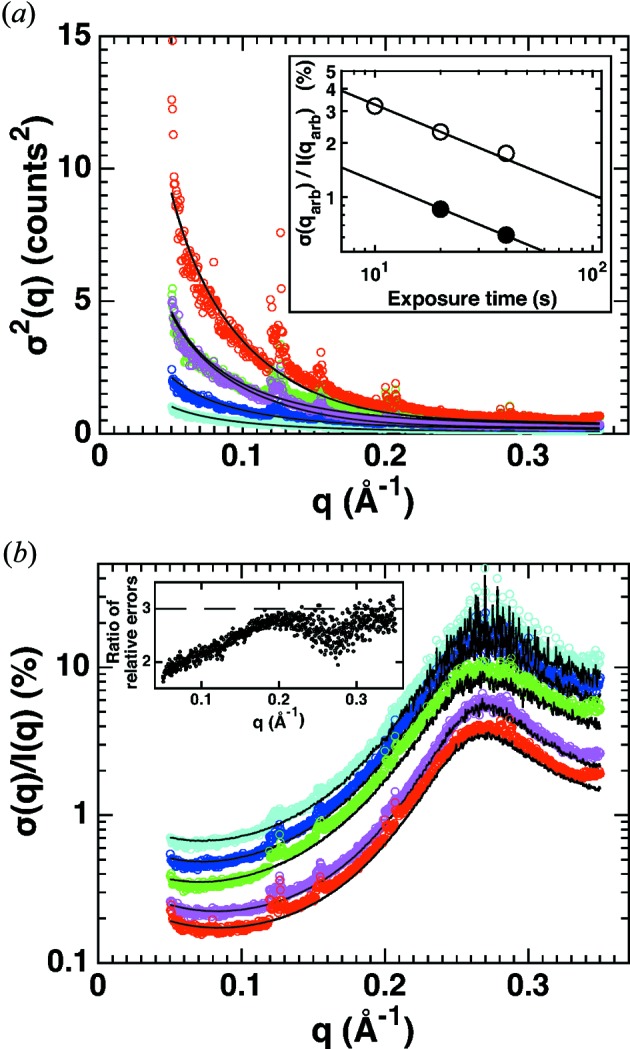
Dependence of SAXS measurement errors on concentration, exposure time and flux. Mean and variances of the buffer-subtracted scattering intensities were determined from repeat exposures under a range of measurement conditions at synchrotron beamline BM29, ESRF, Grenoble (coloured symbols). The absolute variances (*a*) and the relative errors (*b*) are shown. Measurement conditions (protein concentration, number of exposures and length of each exposure) were 8 mg ml^−1^, 5 × 2 s (cyan); 8 mg ml^−1^, 10 × 2 s (blue); 8 mg ml^−1^, 10 × 4 s (green); 24 mg ml^−1^, 10 × 4 s (red); 24 mg ml^−1^, 10 × 4 s at half beam intensity (magenta). Fits of our error model with *c* and *k* as fitting parameters are shown as black solid lines. The fitting parameters are (using *q*_arb_ = 0.2 Å^−1^) *I*(*q*_arb_) = 12.21, *k* = 6134, *c* = 0.8750 (cyan data); *I*(*q*_arb_) = 24.20, *k* = 5873, *c* = 0.8757 (blue data); *I*(*q*_arb_) = 45.77, *k* = 5273, *c* = 0.8737 (green data); *I*(*q*_arb_) = 142.08, *k* = 4822, *c* = 0.6894 (red data); *I*(*q*_arb_) = 75.49, *k* = 5507, *c* = 0.7186 (magenta data). When fitting a straight line to the number of pixels per *q* bin *N*(*q*), a value for *k* of the same order of magnitude is obtained (*k* = 4387; Fig. S5). The inset in panel (*a*) shows the relative error at *q*_arb_ = 0.2 Å^−1^ as a function of the (flux-corrected) exposure time. The solid lines are fits of a relationship α*t*_exp_^−1/2^, which provide an excellent description of the data for both the low- (8 mg ml^−1^; open symbols) and high-concentration (24 mg ml^−1^; solid symbols) data. From the fit we find α(8 mg ml^−1^)/α(24 mg ml^−1^) = 2.7, close to the concentration ratio. The inset in panel (*b*) shows the ratio of relative errors between 8 and 24 mg ml^−1^ data at the same *t*_exp_ (green divided by red data from the main panel), which is generally close to 3 (indicated as the dashed line), but varies with *q*.

To demonstrate the applicability of our error model and to obtain quantitative estimates of the errors in SAXS profiles under a range of conditions, we collected buffer-subtracted scattering profiles for cytochrome *c* at a state-of-the-art synchrotron beamline with varying sample concentrations, exposure times and beam intensities (§2). For this analysis, image frames were stacked to create a single sample and a single buffer image, on which circular averaging was subsequently performed. To determine the experimental errors, we computed the mean and variance of the scattering intensity in each *q* bin [equations (2) and (6); Fig. 5 ▸]. The total scattering intensities differ for varying measurement conditions. Thus, the absolute values of the variances (Fig. 5 ▸ *a*) do not directly reflect the quality of the SAXS data. As a better and more readily interpreted measure of the signal-to-noise ratio, we therefore focus on the standard deviation relative to the scattering intensity (Fig. 5 ▸ *b*) to discuss the scaling of the observed errors with measurement parameters.

For our experimental data, we find the relative error (for a given flux and protein concentration) to only depend on the total (flux-corrected) exposure time *t*_exp_ and to scale as *t*_exp_^−1/2^ (inset in Fig. 5 ▸ *a*). *t*_exp_ can be increased by increasing either the flux, the exposure time per frame or the number of frames. In practice, the exposure time per frame should not be chosen to be too short: for detectors with non-negligible read-out noise (in particular CCD detectors) the count rate per frame should not be too low; for any detector system processing (too) many frames can be cumbersome for data handling and processing. On the other hand, individual exposure times should not be chosen to be too long, either, as otherwise radiation damage might occur within one frame which is difficult to detect.

For a given *t*_exp_, increasing the protein concentration can reduce the relative errors. Indeed, we find a significant reduction of the relative error for all *q* values at higher protein concentrations for our data set, approximately linear in protein concentration. We note, however, that the dependence of the relative error on protein concentration is complex, since changing the protein concentration will not affect the scattering profile of the buffer and the corresponding contributions to the buffer-subtracted profile (Fig. 5 ▸ *b*, inset). The reduction in relative error with increasing protein concentration would suggest always measuring at the highest possible protein concentration. However, in practice, increasing the sample concentration can be challenging or inadvisable, since high protein concentration can give rise to sample aggregation or interparticle interference effects in the scattering profiles (Lipfert *et al.*, 2009 ▸; Jeffries *et al.*, 2016 ▸; Dyer *et al.*, 2014 ▸).

Our results also suggest guidelines to optimize the setup geometry for particular SAXS measurements. If, for example, the focus is on global conformational changes requiring especially low *q* values to determine reliable *R*_g_ values, a long sample–detector distance with a centrally arranged beam is preferable. In particular, for in-house setups with restrictions in beam intensity and detector dimensions, one should consider increasing the number of pixels for the respective *q* bins by positioning the detector accordingly (Fig. 3 ▸).

While the general recommendations for SAXS measurements obtained here are in line with established guidelines, we note that the quantitative analysis of variance can serve as a diagnostic for experimentalists to test and improve their measurements. Importantly, under all conditions investigated here, our model accurately describes the experimental errors with appropriately chosen parameters (Fig. 5 ▸, solid lines; parameters are given in the figure legend; see Fig. S6 and Table S1 for data on additional proteins).

### 3.6. Experimental errors from independent repeat measurements   

So far our analysis has focused on the errors encountered in measurements of a single aliquot of sample solution, albeit consisting of multiple exposures and using properly matched buffer measurements. We note that, while it is good practice to record multiple exposures to check for radiation damage and to carry out control measurements with dilutions of the same sample solution stock to test for interparticle interference and aggregation effects, it is common to use the buffer-subtracted scattering profile from a single aliquot of sample solution in subsequent SAXS analyses. Nonetheless, it is instructive to determine the level of variation encountered in independent repeat measurements (each involving multiple exposures and buffer subtraction) of aliquots drawn from the same stock solution (sometimes called ‘technical repeats’) or even repeat measurements of independently prepared stock solutions (often called ‘biological repeats’) (Krzywinski *et al.*, 2014 ▸). In particular, for high-flux synchrotron sources where the counting errors can be minimized (Fig. 5 ▸), we expect the variability from technical repeats or, ultimately, independent biological repeats to provide a more realistic assessment of the true measurement error.

**Figure 6:**
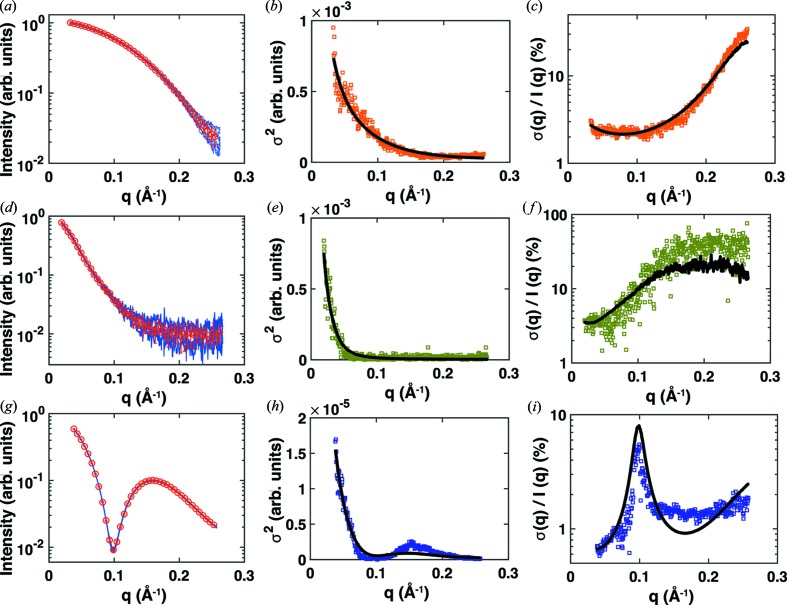
Errors from independent repeat measurements for a range of biological samples. SAXS measurements for 8 mg ml^−1^ cytochrome *c* (*a*)–(*c*), 0.9 mg ml^−1^ full-length wt myosin VI (*d*)–(*f*) and *n*-dodecyl-β-d-maltoside micelles at a detergent concentration of 45 m*M* (*g*)–(*i*). All data were collected at beamline 12ID at the Advanced Photon Source, Argonne, IL, USA, using a CCD detector (Mar CCD165), an X-ray energy of 12.0 keV and a custom-made sample environment (Lipfert *et al.*, 2006 ▸; Beno *et al.*, 2001 ▸; Lipfert, Columbus *et al.*, 2007 ▸; Spink *et al.*, 2008 ▸). The panels on the left (*a*), (*d*) and (*g*) show the individual scattering profiles as blue lines (nine profiles for cytochrome *c*, four for myosin VI and five for dodecyl-maltoside) and the mean and standard deviation for every tenth *q* bin as red symbols and error bars. The middle (*b*), (*e*) and (*h*) and right (*d*), (*f*) and (*i*) panels show the variances and relative errors obtained from the experimental data as symbols and the best fit of the model defined by σ^2^(*q*) = [*I*(*q*) + const.]/(*k* *q*) as black lines.

*A priori*, the biological variability of independently prepared solutions depends strongly on the nature of the sample and method of preparation, which is beyond the focus of this study. Here, we therefore investigate the variability observed in technical repeats using multiple aliquots of sample and buffer solution. For each *q* bin, we computed the mean of and the variance between scattering profiles of technical repeats, which were independently recorded, circularly averaged and buffer subtracted, for a range of biological samples (Fig. 6 ▸). Our data set includes cytochrome *c*, full-length wt myosin VI (Spink *et al.*, 2008 ▸) and *n*-dodecyl-β-d-maltoside micelles (Lipfert, Columbus *et al.*, 2007 ▸) that give rise to very distinct scattering profiles (Figs. 6 ▸ *a*, 6 ▸ *d* and 6 ▸ *g*). We note that, while the variance observed in repeat measurements on different aliquots is still fundamentally constrained by the arguments outlined in the sections above, there can be additional contributions to the errors (*e.g.* variations in fluid handling, sample cells and synchrotron flux between the different measurements) and, *a priori*, one would expect deviations from the error model given by equation (10). Indeed, we observe relative errors from repeat measurements on independent aliquots (Figs. 6 ▸ *c*, 6 ▸ *f* and 6 ▸ *i*) that are higher than the errors seen for measurements on single aliquots (Fig. 5 ▸). Nonetheless, we find that the errors obtained from repeat measurements on independent aliquots are still reasonably well described by the model of equation (10), when considering both the variances (Fig. 6 ▸ *b*, 6 ▸ *e* and 6 ▸ *h*) and the relative errors (Fig. 6 ▸ *c*, 6 ▸ *f* and 6 ▸ *i*). Here, the values of *I*(*q*_arb_), *c* and *k* lose their physical interpretation, so that it is reasonable to condense *I*(*q*_arb_) and *c* into a single constant const. and rewrite equation (10) as σ^2^(*q*) = [*I*(*q*) + const.]/(*kq*). This is the basic functional form of our model, where *k* and const. are treated purely as fitting parameters without any direct physical interpretation. The results (Fig. 6 ▸) suggest that the functional form of our model adequately captures the variability even for technical repeats, which, in turn, implies that our model provides a fairly general description of measurement variability for simulating measurement errors. As our model only takes into account Poisson noise and no other sources of error, this result might be surprising and raises the question of to what extent the deviations between technical repeats are still dominated by counting statistics. For ideal technical repeat measurements (without errors caused by buffer or concentration mismatch or differences in alignment of the setup or the beam) with modern noise-free hybrid pixel detectors, the remaining errors are statistical errors due to counting statistics. This suggests that significant deviations of the variances between technical repeat measurements from the functional form of our model may be used to identify systematic errors, such as buffer mismatch.

### 3.7. Recommendations for simulating errors for theoretical SAXS data   

A number of increasingly powerful analysis techniques have been and are being developed to analyse SAXS data (see §1). Not only do these techniques require a precise treatment of experimental errors, but very often they rely on simulated SAXS data for testing and performance evaluation (Schindler *et al.*, 2016 ▸; Bernadó *et al.*, 2007 ▸; Pinfield & Scott, 2014 ▸; Zettl *et al.*, 2016 ▸). There are several programs to compute SAXS profiles from high-resolution structures (Svergun *et al.*, 1995 ▸; Schneidman-Duhovny *et al.*, 2010 ▸; Poitevin *et al.*, 2011 ▸; Ravikumar *et al.*, 2013 ▸; Chen & Hub, 2015 ▸). However, to simulate realistic SAXS profiles that are representative of the experimental situation, it is important to add errors to the calculated scattering profiles (Rambo & Tainer, 2013*b* ▸). Here, we provide a concrete procedure for simulating SAXS data with realistic errors. Starting from an ideal, error-free SAXS profile computed from a high-resolution structure, we have to first scale the theoretical scattering intensity to a number of counts per *q* bin representative of real SAXS measurements. The best agreement between experiment and modelling is achieved using the exact number of pixels per *q* bin *N*(*q*) to estimate the standard deviation and thus considering the exact measurement geometry (Figs. S7*e* and S7*f*). However, if experimental details are unknown or to generate ‘generic’ yet realistic errors, the approximation stated in equation (10) can be used. Recommended values for synchrotron and in-house measurements are provided in Table 1 ▸.

We recommend the following procedure [a MATLAB (The MathWorks Inc., Natick, MA, USA) code is given in Fig. S8]:

(i) Compute a theoretical scattering profile *I*_t_(*q*) from a crystal structure (using *CRYSOL*, *FoXS* or another program).

(ii) Normalize the scattering profile by dividing it by *I*_t_(0).

(iii) Multiply the scattering profile by a factor of 100 (10) to scale to a realistic number of counts for a synchrotron (in-house) setup.

(iv) Calculate the variance σ_t_^2^(*q*) by equation (10) using *k* = 4500, *c* = 0.85 and *q*_arb_ = 0.2 Å^−1^. [Here, *q*_arb_ is chosen for typical SAXS geometries resulting in a *q* range covering up to *q*_max_ ≃ 0.35 Å^−1^. The values of *k* and *c* were estimated from the experimental data set (Table S1) and can be adjusted to match sample, buffer and setup geometry.]

(v) Compute a scattering profile with errors *I*_e_(*q*) employing a random-number generator and a Gaussian distribution with mean *I*_t_(*q*) and standard deviation σ_t_(*q*).

**Figure 7:**
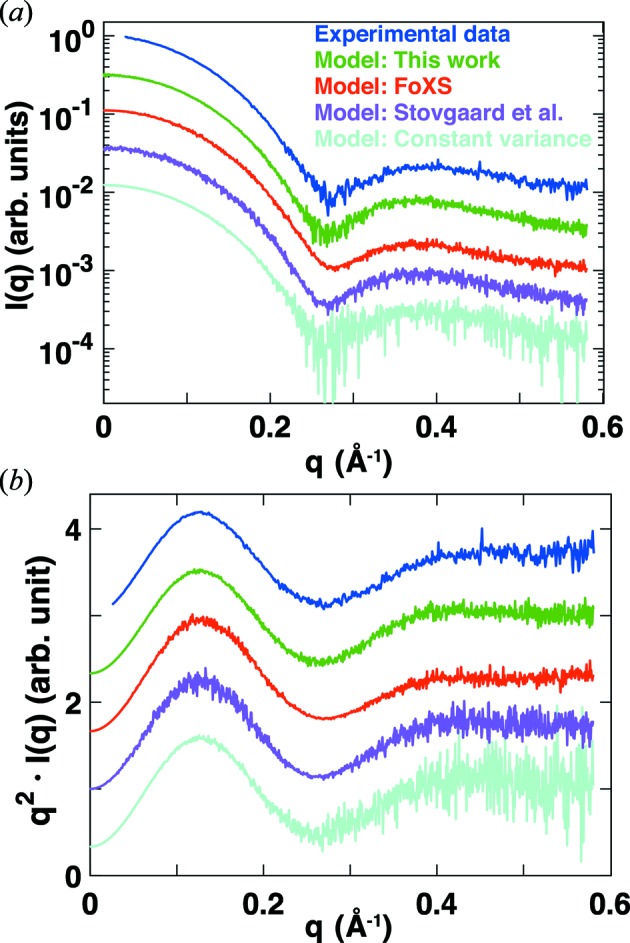
Comparison of experimental data and models for errors in SAXS data. (*a*) Intensity *versus q* and (*b*) Kratky representation [*q* *I*(*q*) *versus q*] of scattering profiles for cytochrome *c*. Experimental data (Lipfert *et al.*, 2006 ▸) for 8 mg ml^−1^ cytochrome *c* in 100 m*M* acetate buffer (pH 4.6) with 0.5 *M* guanidinium hydrochloride added recorded at beamline 12ID at the Advanced Photon Source, Argonne, IL, USA are shown in blue. Simulated scattering profiles were computed using *CRYSOL* (Svergun *et al.*, 1995 ▸) (green, magenta and cyan profiles) or *FoXS* (Schneidman-Duhovny *et al.*, 2010 ▸) (red profile) from the crystal structure (Sanishvili *et al.*, 1995 ▸) of cytochrome *c* (PDB accession code 1crc). Simulated noise was added to the computed profiles using (i) the procedure described in §3.7 of this work (green); (ii) the errors from the *FoXS* webserver (red); (iii) the error model of Stovgaard *et al.* (2010 ▸), σ(*q*) = *I*(*q*)(*q* + α)β with the constants adjusted to α = 0.15 and β = 0.2 (magenta); and (iv) Gaussian noise with a constant variance set at σ = 0.5%*I*(0) (cyan).

We have simulated SAXS profiles with noise added using the procedure outlined above and have found that they closely resemble experimental data and provide a more realistic description compared with previously used error models (Fig. 7 ▸ and Fig. S7).

## 4. Summary   

We have derived a new error model for SAXS data which incorporates the measurement process and the setup geometry, and thereby correctly describes the magnitude and scaling of the measurement errors. We have demonstrated its broad applicability to a range of samples, setups, exposure times and sample concentrations. Moreover, we provide guidelines on how to employ the model to simulate uncertainties and to model realistic noise for theoretical scattering profiles. The theoretical scattering profiles simulated using our protocol closely resemble experimental data and we expect our model to be widely applicable to generate synthetic test data sets for the validation of new SAXS modelling approaches. As our model is based on a few simple and general assumptions, we anticipate that similar arguments can be applied to other techniques that employ hybrid photon couting detectors such as correlated X-ray scattering (Mendez *et al.*, 2014 ▸).

## Supplementary Material

Supporting information file. DOI: 10.1107/S1600576717003077/jo5030sup1.pdf

## Figures and Tables

**Table 1:** Typical values for *k* and *c* for in-house and synchrotron SAXS experiments Typical values (*q*_arb_ = 0.2 Å^−1^).

	*I*(*q*) (in counts)	*k* (in Å)	*c*(*q*_arb_)
Synchrotron	1–100	4500	0.85
In-house setup	0.1–10	4500	0.90
