# Supplementary material for: Quantitative evaluation of statistical errors in small-angle X-ray scattering measurements
Source: J Appl Crystallogr. 2017 Mar 29;50(Pt 2):621–30. doi: 10.1107/S1600576717003077 (PMC5377352; doi:10.1107/S1600576717003077)
Supplement: Supplementary file 1 [file j-50-00621-sup1.pdf]

JOURNAL OF APPLIED CRYSTALLOGRAPHY

Volume 50 (2017)

Supporting information for article:

Quantitative evaluation of statistical errors in small-angle X-ray scattering measurements

Steffen M. Sedlak, Linda K. Bruetzel and Jan Lipfert

S1. Detailed derivation of our error model

Equation 2 follows from the general consideration that for a set of independent measurements, the standard error of the mean is given by

$$\bar{\sigma} = \sigma/\sqrt{N}$$

In our case the counts n_i in every pixel i belonging to the same q-bin are considered as $N(q)$ independent measurements. Since the buffer or sample scattering intensity $I_{s/b}(q)$ for a certain scattering angle q are calculated by taking the mean of the photons recorded by all $N(q)$ pixels belonging to the same q-bin, the corresponding errors $\sigma_{s/b}(q)$ and variances $\sigma^2{}_{s/b}(q)$ can be calculated as

$$\sigma_{s/b}(q) = \sigma_i/\sqrt{N(q)}$$

$$\sigma_{s/b}{}^2(q) = \sigma^2{}_i/N(q)$$

Here, we assumed that all pixels belonging to the same q-bin have the same variance σ_i. As this might not be the case, the best approximation is to average over all variances σ_i, so that

$$\sigma_{s/b}{}^2(q) = 1/N(q)\,1/N(q)\sum_{i=1}^{N(q)}\sigma^2{}_i$$

While we employ this equation to derive our model, we use the expression

$$\sigma_i{}^2 = (n_i - I(q))^2$$

to experimentally determine the variance σ_i^2, i.e. by squaring the difference between the counts in a certain pixel and the mean intensity in the corresponding q-bin:

In the derivation of Equation 8, the following steps are used:

$$I_s(q_{arb}) = I(q_{arb}) + I_b(q_{arb})$$

$$I_s(q_{arb}) = I(q_{arb}) + c\,I_s(q_{arb})$$

$$I(q_{arb}) = I_s(q_{arb}) - c\,I_s(q_{arb})$$

$$I(q_{arb}) = (1 - c\,)\,I_s(q_{arb})$$

$$I_s(q_{arb}) = \frac{I(q_{arb})}{(1 - c)}$$

Table S1 **Experimentally fitted parameters.** Experimentally determined fit parameters for *k*, *c* and $I(q_{arb})$ for different measurement conditions at in-house and synchrotron setups are determined by fitting Equation 10 to the deviations between intensities determined from several frames of sample and buffer measurements on which circular averaging has been performed individually, i.e. the variance between repeat exposures. While *c* and $I(q_{arb})$ are well-defined, the free parameter *k* scatters more broadly, because it incorporates many different parameters (Equation 9).

Experimental fits (q_{arb} = 0.2 Å^{-1})

**Synchrotron** (λ = 0.9919 Å)	conc. (mg/ml)	exp. time (s)	frames	$I(q)$ (counts)	k (Å)	$I(q_{arb})$ (counts)	$c(q_{arb})$	$I_b(q)^*$
Lysozyme	5	1	10	1-100	2756	3.5	0.87	46.8
L_{sd} = 2.864 m	10	1	10	1-100	2593	6.5	0.79	48.9
(multibunch)	20	1	10	1-100	2563	13.0	0.65	48.3
Cytochrome *c*	2	4	10	1-100	4551	1.2	0.97	77.6
L_{sd} = 2.872 m	8	4	9	1-100	4274	4.5	0.87	60.2
(multibunch)	24	4	9	1-100	5520	14.1	0.69	62.8
Cytochrome *c*	2	4	10	0.1-10	6858	0.5	0.97	32.3
L_{sd} = 2.872 m	8	4	10	0.1-10	6327	2.4	0.89	38.8
(low bunch)	24	4	10	0.1-10	5213	7.5	0.72	38.6
**In-house** (λ = 0.7085 Å)	conc. (mg/ml)	exp. time (s)	frames	$I(q)$ (counts)	k (Å)	$I(q_{arb})$ (counts)	$c(q_{arb})$	$I_b(q)^*$
Cytochrome *c*	2	7200	4	0.01-1	2572	-0.17	1.06	6.0
L_{sd} = 1.109 m	8	7200	4	0.1-10	3363	0.48	0.87	6.4
	24	7200	3	1-100	5934	1.27	0.71	6.2
Lysozyme	5	7200	5	0.1-10	3284	0.33	0.92	7.6
L_{sd} = 1.109 m	10	7200	4	1-100	4133	0.59	0.87	7.9
	20	7200	5	1-100	4833	1.12	0.71	5.5
BSA	5	7200	4	0.1-10	3663	0.43	0.91	8.7
L_{sd} = 1.109 m								

Synchrotron data were taken in multibunch mode (170 mA beamcurrent) and low bunch mode (90 mA beamcurrent).

*The buffer level $I_b(q)$ is calculated by $(2\ c\ I(q_{arb}))\ /\ (1-c)$.

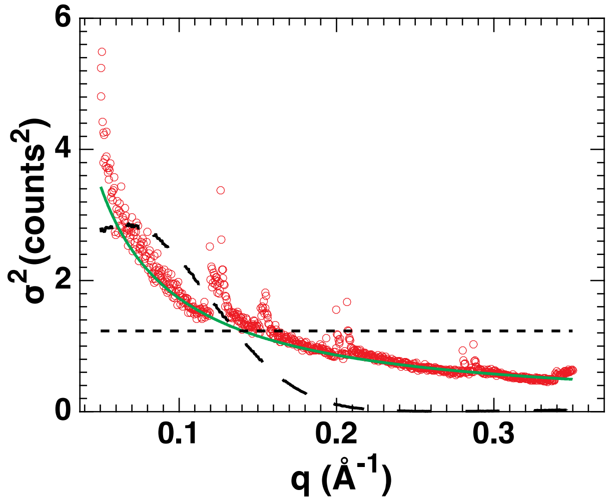

Figure S1 **Comparison of different error models.** Measurement errors obtained from repeat exposures of cytochrome *c* (8 mg/ml, 10 frames of 4 s exposure time each; BM29, ESRF, Grenoble) are fitted with different error models for comparison. The variance of the intensity determined from the 10 exposures is shown as red circles. The model from (Stovgaard *et al.*, 2010) with fitted values α = 0.0520, β = 0.0279 is shown as the black dashed line. The best fitting constant variance σ^2 = 1.232 is shown as a black dotted line. The best fit of our new model with k = 3681, $I(q_{arb})$ = 45.77, c = 0.8737, q_{arb} = 0.2 Å^{-1} is shown as the green line.

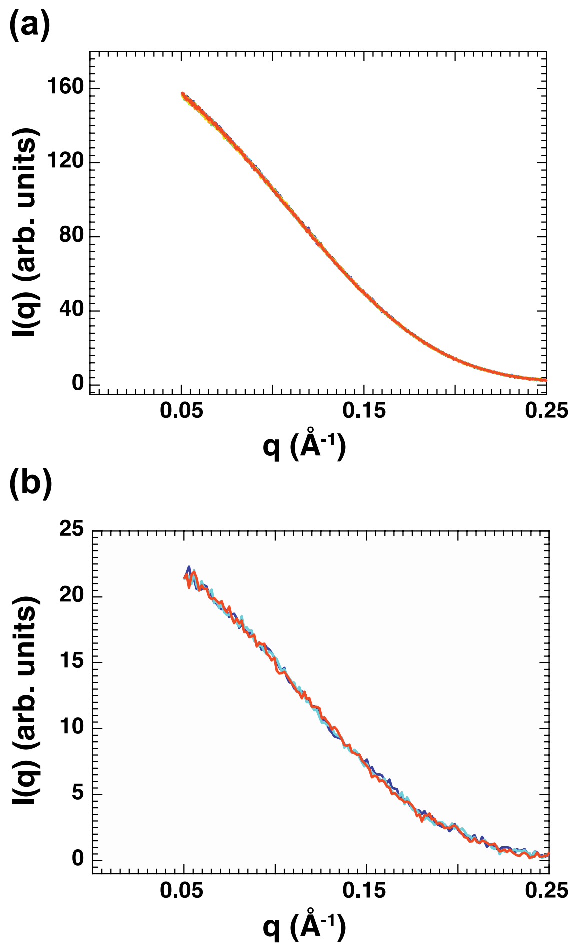

Figure S2 Absence of radiation damage. Overlay of profiles of repeat exposures of cytochrome *c* (24 mg/ml) recorded at (a) a synchrotron source (BM29, ESRF, Grenoble) and (b) at our in-house source (Department of Physics, LMU Munich). No significant differences between the scattering profiles, in particular at small angles, are observed. This confirms the absence of radiation damage. For the other data used in this study the absence of radiation damage was confirmed in the same way.

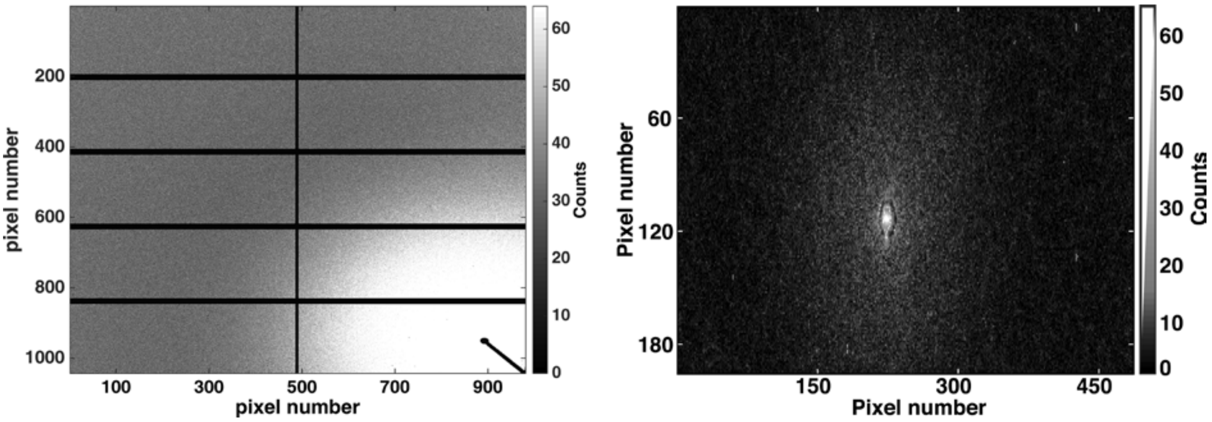

Figure S3 **Typical 2D detector images from synchrotron and our in-house setup.** (a) Scattering pattern of cytochrome *c* (8 mg/ml) recorded at beam line BM29, ESRF, Grenoble. The detector (Pilatus 1M) is built up from ten detector arrays. In between, pixels are missing, resulting in slightly increased measurement errors for certain angles (Figure 2). The shadow of beamstop and beamstop mounting in the lower right corner is masked out. (b) Scattering pattern of cytochrome *c* (8 mg/ml) using a Pilatus 100k detector at an in-house setup at the Department of Physics, LMU Munich. The bright spot and the dark ring in the very middle of the image are caused by the direct beam and the semitransparent beamstop and are masked out when processing the data.

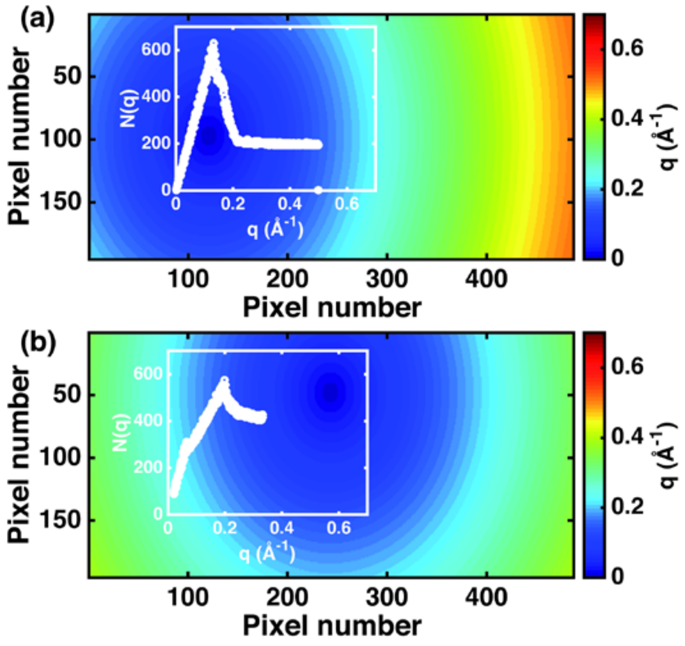

Figure S4 **Assignment of pixels to *q*-bins.** Depending on the position of the beam center on the detector, different pixels are assigned to different *q*-bins. The number of pixels per *q*-bin and the size of the *q*-range can be varied by rearranging the detector position. The corresponding numbers of pixel per *q*-bin *N(q)* are shown in the insets.

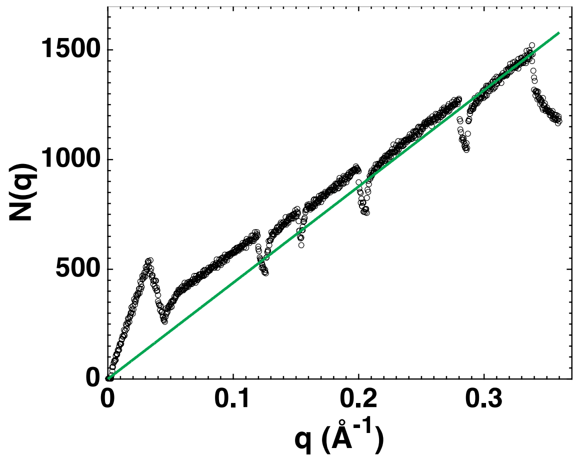

Figure S5 **Number of pixels per *q*-bin *N(q)* for a synchrotron measurement.** The number of pixels per *q*-bin *N(q)* (black circles) for the measurements shown in Figure 5. The detector image is depicted in Supplementary Figure S3a Missing pixels between the different detector arrays result in a decrease of number of pixels *N(q)* for certain scattering angles *q*. The green line is the best fit of the form $N(q) = k\,q$ with $k = 4387$. The value of *k* is in good agreement with the values obtained from fitting the variances (Figure 5).

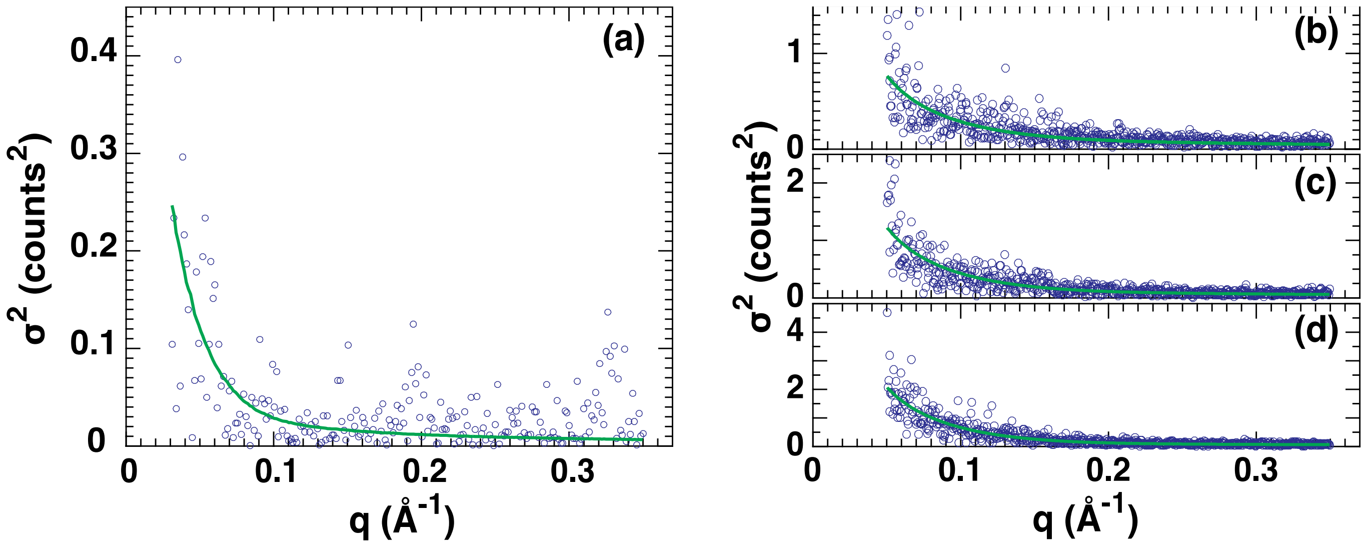

Figure S6 Analysis of SAXS measurement errors for bovine serum albumin and lysozyme. Measurement errors of (a) BSA SAXS data obtained at our in-house setup at the Department of Physics, LMU Munich and (b)-(d) lysozyme SAXS data obtained at a synchrotron beamline (BM29, ESRF, Grenoble) are fitted with our model (similar to the analysis presented in Figure 4 of the main text). Blue circles: variance computed from 4 repeat exposures (of 2 h each) using 5 mg/ml BSA (a) and 10 repeat exposures (of 1.0 s each) using 5 mg/ml (b), 10 mg/ml (c) and 20 mg/ml (d) lysozyme. Green lines: fits using Equation 10. The corresponding fit parameters are listed in Table S1.

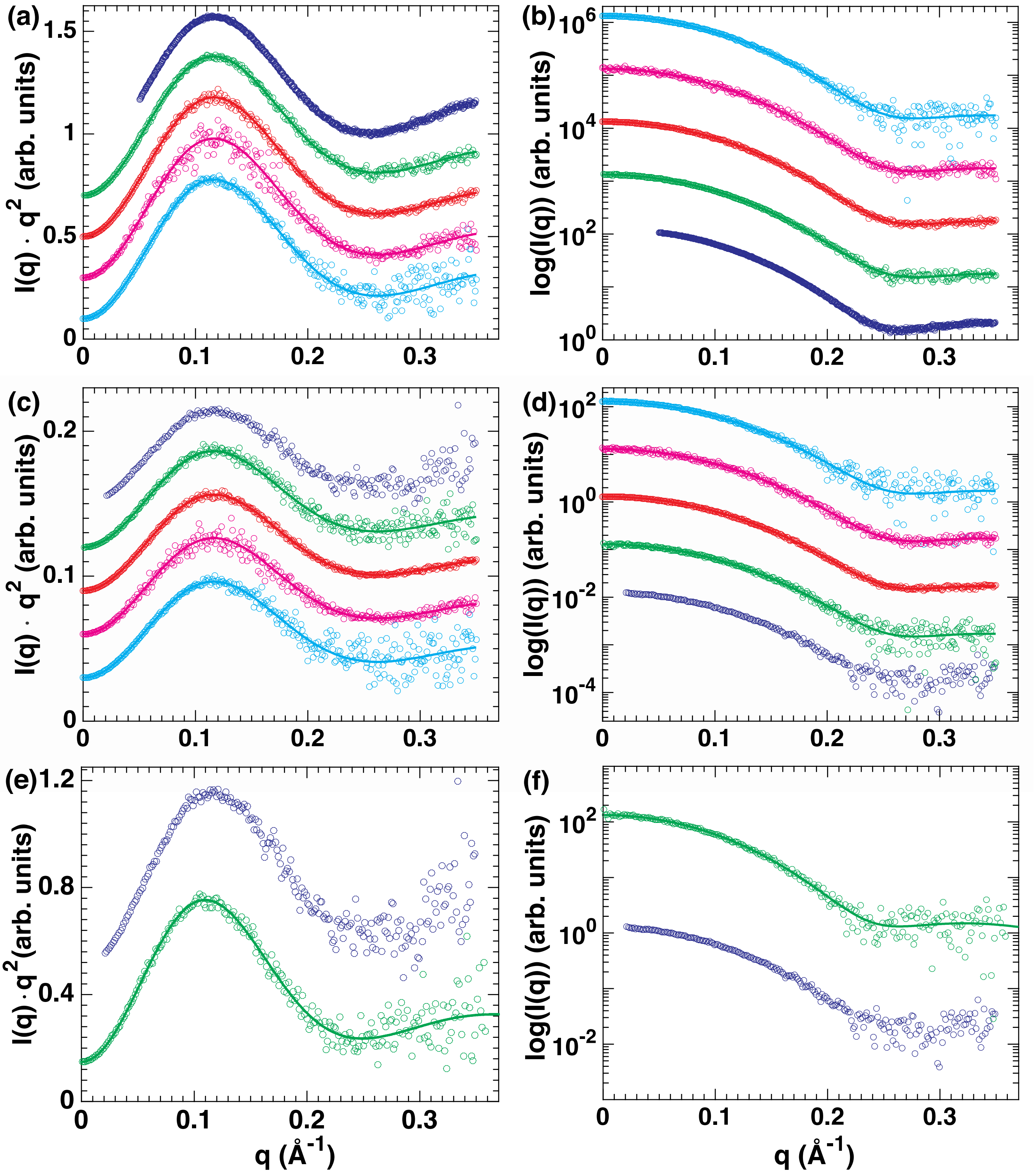

Figure S7 Comparison of noise models for simulated scattering profiles. Simulated and experimental SAXS profiles for lysozyme. Dark blue data in all panels are experimental data measured using 10 mg/ml lysozyme (see Materials and Methods for details). Data in panel (a) and (b) are for synchrotron meaurements (10 frames of 1 s exposure time each; BM29, ESRF, Grenoble). Data in panels (c) - (f) are for in-house data (4 frames of 2 h exposure time each; Department of Physics, LMU

Munich). Panels (a), (c), and (e) show the data in Kratky representation; panels (b), (d), and (f) as log(I) vs. *q*. All profiles are scaled and vertically offset for clarity. The solid lines are theoretical scattering profile computed from the crystal structure (PDB ID: 6LYZ) using FoXS (Schneidman-Duhovny *et al.*, 2010) ((a)-(d)) and CRYSOL (Svergun *et al.*, 1995) ((e) and (f)). The intensities were scaled to mimic experimentally encountered values according the protocol outlined in the main text. The circles color matched to the solid lines are calculated scattering profiles with simulated noise added. They were created by taking a random number for every *q*-bin from a normal distribution with mean $I(q)$ and standard deviation $\sigma(q)$ according to the different error models: 1) Constant standard deviation $\sigma(q) = 0.005 \cdot I(0)$ shown in cyan; 2) Stovgaard's model (Stovgaard *et al.*, 2010) with $\sigma(q) = I(q) \cdot (q + 0.15) \cdot 0.3$ shown in magenta; 3) the variance provided by the program FoXS in red; 4) the new model derived in this work in green. Simulated data using our new model in panel (a) and (b) used $k = 4500$ and $c = 0.85$; simulated data in panel (c) and (d) used $k = 4500$ and $c = 0.90$; simulated data in (e) and (f) used the exact number of pixels per *q*-bin $N(q)$ and $c = 0.90$. The model with constant variance (cyan) tends to underestimate the error at low *q* and/or overestimate the error at high *q*. The model by Stovgaard *et al.* (magenta) and the FoXS model (red) tend to overestimate the errors at intermediate *q* compared to the level of scatter at high *q*.

```
% read in the data so that the scattering angle is
% stored in q and the the scattering intensity in I

k=4500; c=0.85;
I=(100.*I)./(I(1,1);
[~, idx]=min(abs(0.2-q));
Iarb=I(idx);
s=sqrt((1./(k.*q)).*(I+(2*c*Iarb)./(1-c)));
Ie=normrnd(I,s);
```

Figure S8 MATLAB code to simulate realistic errors onto theoretical profiles. Providing the momentum transfer vector *q* and the respective theoretical scattering intensities in a vector *I*, this code can be employed to model realistic noise. The scattering intensities with errors added are stored in the vector *Ie*, the standard error in the vector *s*. The parameters shown are for a typical synchrotron measurement.
